# Use of Double Gelled Microspheres to Improve Release Control of Cinnamon-Loaded Nanoemulsions

**DOI:** 10.3390/molecules29010158

**Published:** 2023-12-27

**Authors:** Esther Santamaría, Alicia Maestro, Carmen González

**Affiliations:** Chemical Engineering and Analytical Chemistry Department, Faculty of Chemistry, Universitat de Barcelona, Martí i Franquès, 1, 08028 Barcelona, Spain; esthersantamaria@ub.edu (E.S.); carme.gonzalez@ub.edu (C.G.)

**Keywords:** encapsulation, double gelled microspheres, nanoemulsions, cinnamon oil, low-energy emulsification method, alginate, chitosan, controlled release

## Abstract

The use of nanoemulsions as encapsulation systems for active ingredients, such as cinnamon oil, has been studied. A surfactant based on polyoxyethylene glycerol esters from coconut/palm kernel oil has been used. The nanoemulsions were obtained by the two most commonly low-energy emulsification methods, the composition inversion phase (PIC) and the temperature inversion phase (PIT) methods. Nanoemulsions were successfully obtained by both methods, with very small droplet sizes (5–14 nm) in both cases, but a greater stability was observed when the PIT method was used. Nanoemulsions were encapsulated by external gelation using two different polysaccharides, alginate or chitosan, dissolved in the continuous phase of the nanoemulsion. Then, the nanoemulsion was dropped into a bath with a gelling agent. To improve the release control of cinnamon oil and avoid the burst effect, beads prepared with one of the polysaccharides were coated with the second polysaccharide and then gelled again. Double gelled beads were successfully obtained, the core with chitosan and the outer layer (shell) with alginate. SEM images showed the morphology of the single beads presenting high porosity. When the beads were coated, the porosity decreased because the second polysaccharide molecules covered the pre-existing pores. The smoother surface was obtained when this second layer was, in turn, gelled. The release patterns at pH = 2 and pH = 7 were studied. It was observed that the double gelled bead provided a more gradual release, but maintained approximately the same amount of final released oil. The release patterns were fitted to the Korsmeyer-Peppas model. The fitting parameters reflected the effect of the different coating layers, correlating with different diffusion mechanisms according to the bead core and shell materials.

## 1. Introduction

In recent years, consumer demand for products enriched with molecules that prevent diseases and increase the added value of products has increased exponentially. This fact has pushed the food industry to develop functional food products incorporating bioactive compounds beneficial to health. There are many classes of molecules that act as bioactive compounds of great interest, such as flavonoids and non-flavonoid phenolic compounds, carotenoids, essential oils, etc. [1,2]. The main challenge in the development of functional foods is the incorporation of these bioactive molecules into food matrices, using edible systems capable of encapsulating, protecting and releasing molecules in a controlled way [3].

In recent decades, the use of natural antimicrobial agents, such as essential oils, has attracted the attention of researchers. It is well described that the antimicrobial activity of essential oils derives from their molecular hydrophobicity [4]. Their low solubility in water makes it necessary to store them in suitable locally hydrophobic carriers dispersed in hydrophilic matrices, from which they can be released to promote their effectiveness [5].

One way to trap essential oils in aqueous matrices is to disperse them in direct nanoemulsions (oil-in-water), which are subsequently encapsulated through gelation of the continuous phase. In this way, the essential oil is incorporated and protected, and its release rate can be controlled. Due to their ability to disperse oils in the aqueous phase, nanoemulsions are perfect systems for incorporating these active ingredients [6]. Different researchers have studied nanoemulsions using essentials oils as the main active compound, such as carvacrol [7,8], eugenol [9], lemon oil [10], and cinnamon oil [11], among others. In the present study, cinnamon oil, from *Cinnamon zeylanicum*, is used due to its excellent properties. Among its qualities are antifungal, antiviral and antibactericidal properties [12]. Moreover, it is used in the treatment of cardiovascular diseases because it reduces blood glucose, as well as cholesterol [13].

Nanoemulsions are a type of emulsion with a very small mean droplet size, normally less than 200 nm, and, as emulsions, they are thermodynamically unstable systems that tend to separate over time into two phases. Due to their small droplet size, these emulsions have a transparent or slightly bluish appearance, and their destabilization can be very slow. Therefore, they are perfect candidates for use in foods to avoid coloration and an undesired appearance or taste to consumers, and to have a reservoir of the active principle with a sustained release. As non-equilibrium systems, nanoemulsions require the application of energy to be formed. Depending on the way this energy is introduced, the formation mechanisms of nanoemulsions are divided into high-energy mechanisms, where high mechanical shear forces are used, and low-energy mechanisms, where chemical energy and changes in spontaneous curvature during emulsification are used instead, under gentle agitation. Many works on the use of nanoemulsions in the food industry use high-energy mechanisms to obtain very small diameters, although low-energy methods are usually preferred by the industry [14,15,16,17,18,19,20].

Low-energy methods are divided into three types: (i) the phase inversion temperature method (PIT), (ii) the phase inversion composition method (PIC), and (iii) spontaneous emulsification. In this article, cinnamon oil nanoemulsions were obtained by the first two methods. The PIT method was introduced by Shinoda [21] and is based on the change of interface spontaneous curvature due to the change in hydrophilicity of surfactants with ethylene oxide groups when the temperature is changed. A mixture is prepared at a temperature higher than the phase inversion temperature of the system, related to the hydrophilic–lipophilic balance temperature (HLB), the temperature at which the preference of the surfactant by the aqueous and organic phases is the same. When this mixture is suddenly cooled, the ethylene oxide chains are hydrated and a spontaneous change in curvature occurs at the interface, from negative or zero to positive. The macroemulsion inverts and goes from a water-in-oil emulsion to an oil-in-water emulsion with a very small droplet size. In order to establish this mechanism, it is important to know the hydrophilic–lipophilic balance (HLB) temperature of the system [11,22].

The PIC method, used by some authors [7,8,23,24], consists of changing the spontaneous curvature of the interface by the progressive addition of a component or a mixture of components; in this case, starting with an oil–surfactant mixture and adding water or water plus cosurfactant until the spontaneous curvature changes and it changes from a water-in-oil emulsion to an oil-in-water fine emulsion.

The encapsulation of active compounds in polysaccharide spheres, such as alginates, pectins, chitosan and other biopolymers, allows the introduction and release of these compounds in a slow and sustained manner in the receiving medium. Some studies have shown that some polysaccharides, such as alginate (Alg), are quite porous [25] and the release of the active molecules encapsulated inside is very fast. Some authors have reported that a mixture of polysaccharides improves the release control and protection of the active compound [8,25,26].

The objective of the present study is to encapsulate direct nanoemulsions with cinnamon oil in different core matrices and evaluate the possibility of controlling the release rate by adding another layer of polysaccharide (shell), which generates an additional resistance to diffusion but allows the active compound to pass through. The beads are designed to enrich foods and preserve them, such as in sauces and desserts. In this way, the release of the active ingredient is evaluated at acidic and basic pHs, which are the most common pHs of foods.

## 2. Results

### 2.1. Phase Diagram

**Figure 1:**
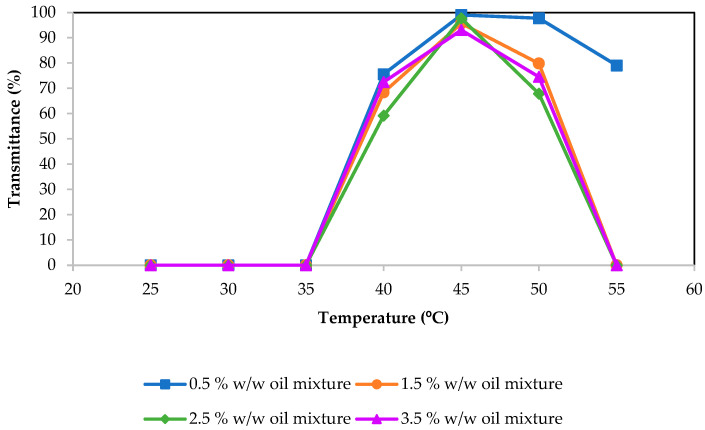
Transmittance values at different temperatures of mixtures with 10% *w*/*w* of surfactant, oil mixture (30/70 cinnamon oil/olive oil) and water.

In order to understand the phase behavior of the system and, therefore, know in which conditions and compositions nanoemulsions can be obtained by low-energy methods, it was decided that the phase diagram at HLB temperature and room temperature (25 °C) would be studied. The final composition of the obtained nanoemulsions was set at 10% *w*/*w* of surfactant according to Chang et al. [27], who reported that a 10% *w*/*w* of surfactant was a good balance between the amount used and, therefore, the cost of the production and the stability of the nanoemulsions obtained. The ratio of essential oil/carrier oil was set at 30/70 according to previous studies [8] that proved it to be the best ratio in terms of mean droplet diameter and stability. The HLB temperature of a system composed of at least water, an ethylene oxide surfactant and an oil can be defined as the temperature at which no preference of the surfactant by water or oil is observed; therefore, flat surfactant layer structures are obtained, resulting in the formation of bicontinuous microemulsions or lamellar liquid crystals. At this temperature, the intersolubility of water and oil is maximum. According to Chuesiang et al. [11], an approximation of the HLB temperature, the PIT temperature (phase inversion temperature), can be determined as the temperature at which the system has low turbidity due to the formation of a bicontinuous microemulsion. In the PIT temperature determination of the present system, the method of Chuesiang et al. [11] was modified and the turbidity was measured using transmittance sweeps at different temperatures instead of absorbance measurements through spectrophotometry. Figure 1 shows the transmittance of different samples vs. the temperature. It can be seen that the transmittance of the samples, which indirectly measures their transparency, increases from 35 °C to 45 °C, which is the maximum for the four oil compositions studied. At 45 °C, the samples show quite a high degree of transparency, indicating a maximum of intersolubility, so it seems to be a good approximation of the HLB temperature of the system. It should be said that, in any case, the sample was completely transparent. Because the temperature of maximum transparency was 45 °C, it was decided that the phase diagram would be studied at this temperature.

**Figure 2:**
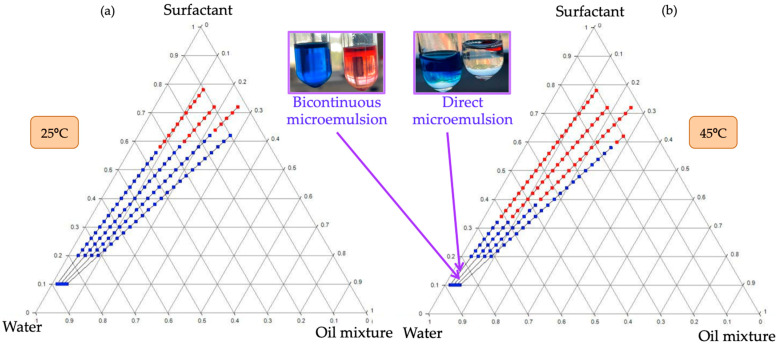
(**a**) Phase diagram at 25 °C for a cinnamon/oil 30/70 in the mixture oil and (**b**) phase diagram at 45 °C for a cinnamon oil 30/70 in the mixture oil. Red points correspond to a single-phase region and blue points are multiphasic regions.

Once the PIT temperature was determined, the phase diagram was studied at the PIT temperature (45 °C) (Figure 2a) and at the final temperature of the nanoemulsions (25 °C) (Figure 2b).

Red points show single-phase regions, while blue points indicate multiphasic regions. The multiphasic zones could not always be analyzed in detail because the upper phase of some tubes had a very low amount, separation was very difficult and, consequently, it was difficult to analyze it without contamination. In the areas of one phase, bicontinuous phases were observed in most cases. These phases could be identified using a hydrophilic dye (Brilliant Blue G) and a hydrophobic dye (Sudan IV). The use of these dyes established the nature of the continuous domain of the sample. A pair of tubes for each chosen point of the diagram was prepared, and a small amount of Brilliant Blue G was accurately located on the surface of one tube, and Sudan IV was located on the other tube. Both tubes were kept at rest, avoiding any type of perturbation, in order to observe the penetration of the dye through the sample only due to diffusion. For each studied point of the phase diagram, if the hydrophilic dye diffused and the hydrophobic dye did not, it indicated that the continuous domain was aqueous and, therefore, a direct microemulsion was present. On the other hand, if the hydrophobic dye was the one that diffused and the hydrophilic dye did not, the continuous domain was oily and an inverse microemulsion appeared. If both dyes diffused, both found a path to penetrate, indicating a bicontinuous nature of the microemulsion (Figure 2).

Figure 2 shows that, at 45 °C, the single zone extends to lower amounts of surfactant and higher amounts of oil mixture, indicating that, at this temperature, the affinity of the surfactant for the oil has increased and less surfactant is required for solubilization, due to the dehydration of the ethylene oxides present in its chains. At this temperature, there is a predominance of bicontinuous structures, corresponding to the HLB temperature. This affinity decreases when the temperature is decreased to 25 °C.

Two phases are present for both temperatures at the composition where the nanoemulsions are prepared (10% surfactant). However, the nature of the phases is not the same. A big amount of sample was prepared, with a composition of 10% *w*/*w* surfactant, 2.5% *w*/*w* oil mixture and 87.5% *w*/*w* water at both temperatures in order to be able to separate the two phases formed, as one of them was very scarce, as said. Using the method of the dyes, the two phases present were identified at 25 °C as direct microemulsion (the predominant one) and a small amount of excess oil phase. However, at 45 °C, most of the sample formed a bicontinuous phase, and the residual separated phase was a direct microemulsion. This behavior is in concordance with the change in the hydrophilic–lipophilic affinity of the surfactant with temperature.

It is well known that the PIT method [21] occurs when, starting from a single phase with a planar structure at a certain temperature, the sample is suddenly cooled, changing the spontaneous curvature of the surfactant layer to a positive one. Very small droplets are then formed that entrap the oil phase, which was initially intimately distributed inside the single planar structure. Other studies [22,28,29] state that the incorporation of all the oil into the initial planar structure is required, so that it is already finely distributed and can be trapped in small droplets when cooling, thus forming nanoemulsions, and the presence of excess of water or direct microemulsion at the HLB temperature does not affect results. What is really relevant is that a free oil phase does not appear at the HLB temperature to be able to prepare proper nanoemulsions. The phase diagram shows that, at 45 °C, there is still a multiphasic zone (Figure 2b), but the dye method indicated that these phases were bicontinuous (most) and direct aqueous phases. That is why nanoemulsions could be formed through PIT from 45 to 25 °C, although no single phase appeared at the HLB temperature. The small amount of aqueous, direct microemulsion phase acted just as a dilution media, as the continuous phase of the nanoemulsions is in fact a direct microemulsion [7,30].

### 2.2. Nanoemulsions Characterization

**Figure 3:**
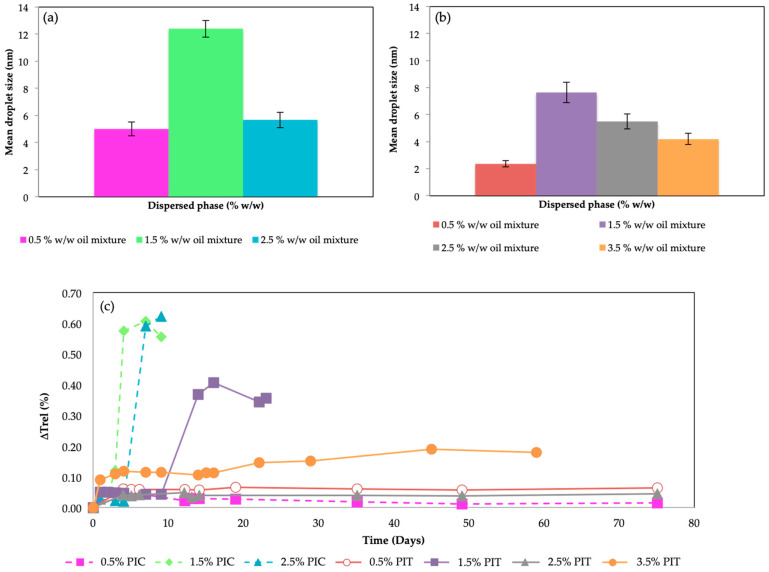
(**a**) Nanoemulsion mean droplet size obtained by the PIC method for different *w*/*w* oil mixtures, (**b**) nanoemulsion mean droplet size obtained by the PIT method for different *w*/*w* oil mixtures and (**c**) change of relative transmittance values for all the prepared nanoemulsions vs. time for different oil mixtures. All samples were prepared with a 10% *w*/*w* of surfactant at the final composition. The relationship of cinnamon oil/olive oil was kept constant at 30/70. Measurement and storage temperature was 25 °C.

The nanoemulsions were characterized as detailed in Section 3.5 and Section 3.6. Figure 3 shows the diameter and stability of the nanoemulsions obtained by both preparation methods, PIT and PIC.

The diameters obtained by both methods were very similar and very small (Figure 3a,b). For the PIC method (Figure 3a), it was not possible to obtain a stable nanoemulsion with a 3.5% *w*/*w* oil mixture. For the PIT method (Figure 3b), nanoemulsions were also obtained with a 4.5% *w*/*w* oil mixture, but they quickly destabilized; only up to 3.5% *w*/*w* were stable enough to be taken into account for encapsulation purposes. The diameters obtained were very small, with a maximum of 14 nm. These diameters are more typical of microemulsions, although it is not the first time that such small diameters have been reported for nanoemulsions; for example, Salvia-Trujillo et al. [31] obtained nanoemulsions of 4.3 nm using high-energy emulsification methods, such as sonication and microfluidization. Sugumar et al. [32] reported nanoemulsions with mean droplet sizes of 3.8 nm using sonication as well. However, to our knowledge, such small diameters have not been reported for low-energy emulsification methods. This confirms that the dispersed systems formed in this work were nanoemulsions and not microemulsions. On the one hand, when all the components were mixed at the same time with a vibromixer, without any method (neither PIC nor PIT), milky macroemulsions were formed that quickly destabilized; therefore, their formation was not spontaneous, which is required for thermodynamically stable systems like microemulsions. On the other hand, Figure 3c shows that not all the nanoemulsions prepared with the same final composition were stable over time, i.e., their stability depended on the emulsification method. To study the stability of the nanoemulsions, the relative change of transmittance over time was studied (Figure 3c). A change in the transmittance would indicate a change in the droplet size or the presence of sedimentation. Therefore, if the transmittance of the samples remained constant over time, it indicated that these emulsions were stable. The nanoemulsions obtained by the PIC method were, in general, less stable than those obtained with the PIT method, except for the most diluted emulsion tested, with only 0.5% *w*/*w* of oil phase, which was very stable for both methods. It seems, therefore, that for the system studied, the PIT method was more suitable than the PIC one. This was related to the fact that the surfactant used was a nonionic surfactant with polyethylene oxide (PEO) and, due to the strong dependence of hydration degree of PEO chains with temperature when the PIT method was used, strong and fast changes in interface spontaneous curvature occurred while cooling, trapping excess oil more properly. Such strong and abrupt changes could not appear by gradual addition of water (PIC method). The only nanoemulsion that presented good stability by the PIC method was the one that used a very low percentage of oil. In that case, the methodology chosen did not impact as strongly as in the case of containing more oil.

### 2.3. Encapsulation of Nanoemulsions

The nanoemulsions prepared were encapsulated as described in Section 3.7. A 2.5% *w*/*w* of oil phase was chosen, prepared by PIT, because this nanoemulsion had a good stability, providing a good percentage of cinnamon oil.

Nanoemulsions were encapsulated in two types of polysaccharide matrix, i.e., in alginate (Alg) and chitosan (Ch), by the dissolution of the corresponding polysaccharide in water and the use of this solution as the continuous phase of the nanoemulsion. The beads were prepared by dropping the nanoemulsion in a solution of the proper gelling agent (1.0% *w*/*v* CaCl_2_ for Alg and 5% TPP for Ch). These beads were then subjected to different types of incubation in order to study the effect of the addition of coatings and gelled layers (core-shell structures) on the release control. It has been reported in numerous studies [8,26,33,34,35,36] that some encapsulation materials, especially alginate, have high permeability, high porosity and poor mechanical properties that reduce the protection of the active ingredient and the ability to control its release. In order to improve these properties, both core beads (Alg and Ch) were firstly formed and subsequently covered by a layer (shell) of the other polysaccharide and, in the case of the Ch-Alg capsules, the shell of alginate was gelled, obtaining a double gelled bead. For the Alg-Ch capsules, the Ch shell could not be gelled using TPP and the double gelled layer was not obtained in an analogous way to Ch-AlgCaCl_2_. When the Alg-Ch beads were immersed in the TPP solution, they disintegrated. As previously described by other authors [37,38], the chitosan molecules remain attached to alginate through the union of the -COO^−^ groups of the alginate with the -NH_3_^+^ group of the chitosan, obtaining beads called Alg-Ch. If these capsules were incubated in a TPP solution, the TPP molecules had the ability to form very strong bonds with chitosan and somehow drag the chitosan molecules from the beads, breaking the -COO^−^ bonds with NH_3_^+^ and disintegrating not only the layer but also the whole bead.

**Figure 4:**
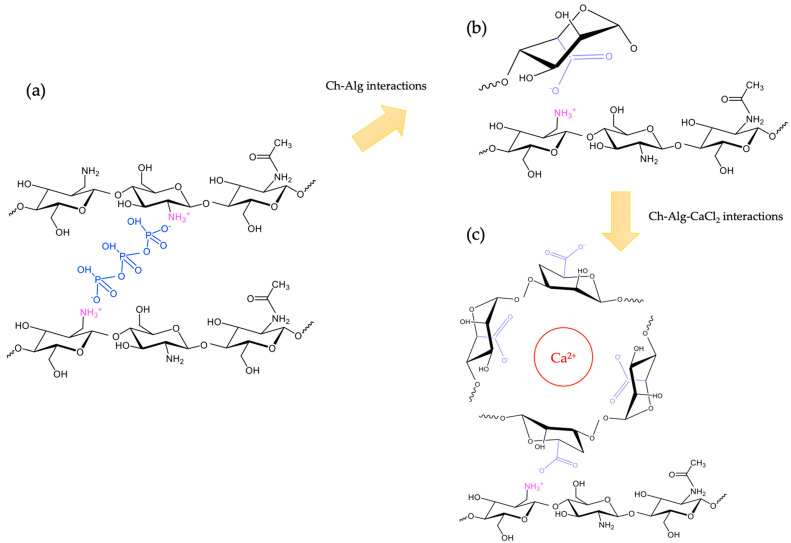
(**a**) Molecular interactions in the chitosan-TPP gelation mechanism, (**b**) molecular interactions between chitosan and alginate coated beads and (**c**) molecular interactions between chitosan and gelled alginate beads.

In the case of Ch-core beads, after the nanoemulsion with the Ch solution as the continuous phase was dripped into a TPP solution, beads called Ch were formed, establishing the links shown in Figure 4a.

The -NH_3_^+^ groups of chitosan formed bonds with the phosphate groups. When these beads were subsequently immersed in an alginate solution to form the shell, the COO^—^NH_3_^+^ bonds (Figure 4b) were formed and the Ch-Alg beads were obtained. In order to gel the shell of alginate, the capsules were then incubated in a 1.0% *w*/*v* CaCl_2_ solution, so that the alginate molecules that were attached to the chitosan formed a second layer gelled by calcium ions (Figure 4c). All beads presented an encapsulation efficiency of 82% (*p* < 0.05) and a cinnamon loading capacity of 2% (*p* < 0.05).

**Figure 5:**
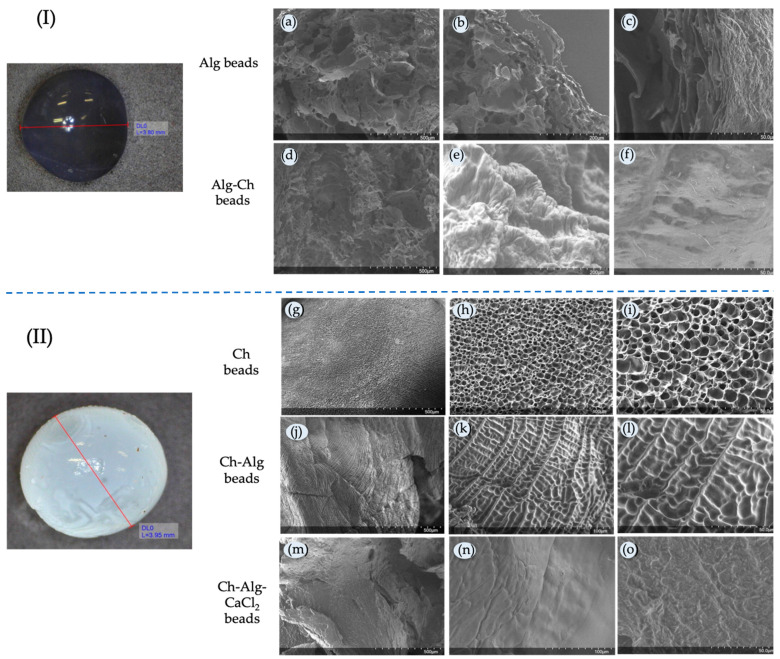
SEM images at different magnifications: (**I**) Alginate based beads: Alg beads (**a**–**c**), Alg-Ch beads (**d**–**f**). (**II**) Chitosan based bead: Ch beads (**g**–**i**), Ch-Alg beads (**j**–**l**) and Ch-Alg-CaCl_2_ beads (**m**–**o**).

Figure 5 I shows the image of alginate beads through the optical microscope, while Figure 5 II shows the chitosan beads. One hundred beads of all types were measured. The beads obtained had an average mean diameter of 3.9 ± 0.1 mm in all cases (*p* < 0.05), measured microscopically, for both chitosan and alginate beads, although the preparation methods were different. No significant differences between the mean values were observed for the uncoated, coated and double gelled beads. They presented different appearances. In the case of alginate-based beads, they had a transparent appearance and, in the case of chitosan, their color was milky white. The morphology of the beads is shown in Figure 5 at different magnifications. The Alg-based beads, i.e., Alg beads and Alg-Ch beads, are shown in Figure 5a–f. The Ch-based ones are shown in Figure 5g–o. It can be seen that, for the Alg single beads, the porosity of the surface was considerable, with big pores (Figure 5a–c), wider than that of the Ch single beads (Figure 5g–i). When Alg beads were covered by a layer of chitosan, forming Alg-Ch beads, the porosity of the beads decreased, as seen in Figure 5d–f, where a smoother surface can be observed. The pores were partially covered by the molecules of Ch adhered to the surface. In the case of Ch-based beads, the single Ch ones had an initial porosity, with smaller and more uniform pores than those presented by Alg; however, when the alginate molecules were introduced as a shell, the images clearly show how the pores were covered. Despite the Alg coating, the porous structure can still be glimpsed through the thin coating layer (Figure 5j–l). The deposition of Alg molecules did not modify the existing porosity but simply covered it, creating a thin layer that would serve as additional resistance to the release flow of the active molecules encapsulated inside. When the Alg coating was gelled, the morphology shown in Figure 5m–o was obtained. In this case, the surface of the beads did not show any pores, and a double gelled bead was obtained.

### 2.4. Cinnamon Oil Release Kinetics

**Figure 6:**
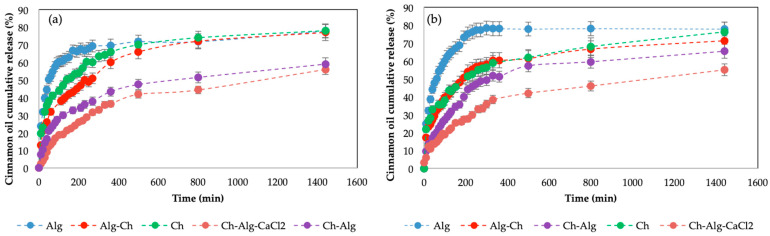
Cumulative cinnamon oil release for Alg beads, Alg-Ch beads, Ch beads, Ch-Alg beads and Ch-Alg-CaCl_2_ beads at (**a**) pH = 2 and (**b**) pH = 7. T = 25 °C.

In order to evaluate the suitability of the beads to delay the release of cinnamon oil at different pH conditions (pH = 2 and pH = 7), the in-vitro release pattern was studied in different buffer solutions at 25 °C. Figure 6 shows the cumulative release patterns of the different prepared beads. Release studies were performed in duplicate to evaluate the reproducibility of the results. It can be seen that the faster release occurred for single beads, that is, those formed just by Alg or Ch, for both pHs. For Ch beads, the release was slower than for Alg beads, corresponding to the lower porosity of the matrix. The addition of a coating to the bead decreased the release rate. For the case of pH = 2 (Figure 6a), it can be seen how the Alg beads, Alg-Ch beads and the Ch beads released the same amount of cinnamon oil at the end of the experiment; however, at short times, the Ch beads and, especially the Alg-Ch beads, released more slowly than Alg ones, making the dosage of cinnamon oil more gradual than in the case of uncoated beads. When Ch-Alg beads and Ch-Alg-CaCl_2_ beads were used, the release was much slower and continued after 24 h, as the slope of the curve was positive and a constant, plateau value was not achieved. For pH = 7 (Figure 6b), a similar behavior was observed, in which it was more clearly seen that the double gelled beads had the slowest release, maintained in a sustained manner for much longer. These results suggest that the shell adds an extra resistance to diffusion of the active principle, especially if the shell is gelled, leading to a more controlled release. During the release processes of active molecules, it is usual that the burst effect occurs [39]. This effect consists of a rapid release of the molecules into the medium. When the molecules have been encapsulated in polysaccharide beads, it is easy for a significant concentration to remain in the most superficial area of the sphere. When the beads are introduced into the release medium, these molecules from the outer layers are quickly released, causing a very rapid increase in concentration known as the burst effect, which is magnified when the gel is porous. It can be seen that the use of a shell can reduce this burst effect, especially if it is subsequently gelled.

To evaluate the diffusion mechanisms involved in the release of cinnamon oil, the experimental values were adjusted to the Korsmeyer-Peppas model (Equation (3)).

Table 1 shows the values obtained for each of the experiments carried out. The Korsmeyer-Peppas model [40] provides information about the diffusion mechanism of the active compound from the encapsulation system to the release medium. For *n* values < 0.45, or 0.43 in the case of the spherical encapsulation shape [41], the drug transport release is considered to be quasi-Fickian diffusion, while for *n* values of ~0.45 or 0.43, the transport should be considered to be Fickian diffusion [41,42,43]. M_24h_ in Table 1 shows the total amount of cinnamon oil released after 24 h, corresponding to the experimental data showed in Figure 6. As previously commented, for 24 h not all the coated or gelled beads completed the release process, so a measurement was made after 4 days in order to obtain a value of M∞ to fit the model to the experimental data. All the different types of beads released the 78% ± 2 of cinnamon oil after 4 days. According to Ferrero et al. [42] and Fu et al. [44], in order to use the semi-empiric power law proposed by Korsmeyer and Peppas, the release data should be fitted until Mt/M_0_ ≤ 0.60, where M_0_ is the initial mass of the cinnamon oil in the beads. It is for this reason that adjustments were made to only the amount released during the first 24 h. These values ranged between 55% and 78% of the initial amount of cinnamon oil present in the beads, which, as can be seen, exceeded this range in some cases, although similar parameters were found if the range was limited to 60%. It was decided that the amount released over a long time would be analyzed in order to verify that the coating layers and double gelation did not prevent release over infinite time, and that the total release was comparable to the single beads.

Table 1 shows that, for beads formed with a single material in the matrix, relatively low *n* coefficients were obtained, 0.1664 for the case of Alg beads and 0.1870 for Ch beads, with a much higher K value than that obtained with double-coated beads. This indicates a fast release, with a low resistance to diffusion and a strong burst effect. When a layer was added covering the spheres, although the links joining core and shell were relatively weak, the coefficient *n* increased to approximately 0.30 for both cases, indicating that it was increasingly approaching a Fickian-type diffusion, and K significantly decreased, corresponding to slower diffusion. In the case of double gelation, the *n* coefficients were already close to 0.40–0.44, and K was much smaller, indicating that this layer acted as a second resistance for the diffusion of the molecules to the medium.

## 3. Materials and Methods

### 3.1. Materials

Food-grade Cinnamon oil (W229202) with a purity ≥98% was purchased from Sigma-Aldrich. The non-ionic surfactant Levenol C-201 was kindly donated by KAO Chemicals. The surfactant Levenol C-201 has a glycereth chain with 17 cocate groups, resulting in a surfactant with a HLB number ~13. Olive oil (Borges brand) with an acid degree 0.4° was purchased from a local supermarket. Chitosan (419419) with >75% deacetylation degree and high molecular weight, M_W_ ≈ 3.1–3.75 × 10^5^ g/mol, anhydrous calcium chloride (C1016) with a granularity ≤7 mm and purity ≥93%, trisodium trimetaphosphate (TTP) (T5508) (M_W_ = 305.89 g/mol) with purity ≥95%, Brilliant Blue G as a hydrophilic dye and Sudan IV as a hydrophobic dye were provided by Sigma-Aldrich. Technical-grade sodium alginate (Alg) with a ratio of β-D-mannuronic acid: α-L-guluronic acid = 58.9:41.1, measured using nuclear magnetic resonance (DMX-500, 500 MHz, Bruker, Billerica, MA, USA), and M_n_ ≈ 668,000 and M_W_ ≈ 1,750,000, obtained using size-exclusion chromatography as described in a previous work [8], was purchased from Panreac.

### 3.2. Determination of Phase Diagram

In order to determine the phase diagram of the system, several mixtures were prepared at different compositions. For the preparation of the selected composition, the components were weighed in a tube, mixed, sealed and left for equilibration in a controlled thermostatic bath at the desired temperature. Once equilibrium was reached (at least 1 week after), the phases present in the tube were evaluated.

### 3.3. Nanoemulsion Formation by PIC Method

In order to obtain nanoemulsions by the PIC method, all the required oil mixture and surfactant were weighed in one tube, while the total amount of water was weighed in another tube. Once the tubes were prepared, a small amount of water from the water tube was transferred to the oil/surfactant tube and mixed by a vibromixer. Both tubes were left in a thermostatic bath at 25 °C for 20 min to ensure that the phase inversion process was carried out at a constant temperature. After 20 min, the remaining water was added drop by drop to the mixture, controlling the temperature, to obtain the final weight composition of the nanoemulsion. After each addition of water, the tube was mixed and allowed to rest in the thermostatic bath for 10 min.

### 3.4. Nanoemulsion Formation by PIT Method

In order to obtain nanoemulsions by the PIT method, all the components were weighed in a tube and mixed with the help of a vibromixer. Milky white macroemulsions were formed. Once the emulsion was prepared, the tubes were heated to the HLB temperature, approximately 45 °C. At this temperature, the emulsion changed its appearance from white to transparent bluish color in approximately 10–15 min. Once the temperature was equilibrated, the sample was mixed in the vibromixer for 10 s and quickly cooled in a cold finger bath (T = 4 °C), while continuing shaking the tube by hand. The tube increased its transparency, indicating that the nanoemulsion was formed.

### 3.5. Droplet Size

The mean droplet size of the nanoemulsions was measured by a 3D dynamic light scattering (3DDLS) spectrometer (LS Instruments, Fribourg, Switzerland) equipped with a He-Ne laser (λ = 632.8 nm) at a scattering angle of 90° and 25 °C. The mean droplet size described in this work was obtained by a cumulant analysis of at least three independent measurements.

### 3.6. Stability Measurements

The stability of the emulsions was determined by studying the variation in the transmittance (T) light (173°) using a Turbiscan Classic MA 2000. The T values were normalized as: ∆T_rel_ = [(T_t_ − T_t0_)/T_t0_]·100, where T_t0_ is the transmittance at time = 0, and T_t_ is the transmittance at time = t. Normalization was done in order to compare changes with time independently from their initial values.

### 3.7. Encapsulation of Nanoemulsions

#### 3.7.1. Encapsulation in an Alginate Matrix

The Buchi Encapsulator B-390 was used for the encapsulation of nanoemulsions into beads. Nanoemulsions with 30% *w*/*w* of cinnamon essential oil in the oil mixture and 2.5% *w*/*w* of total oil mixture were prepared using the PIT method. The final composition of encapsulated nanoemulsions was set to 2.5% *w*/*w* of oil mixture, 10% *w*/*w* of surfactant mixture and 87.5% *w*/*w* of alginate aqueous solution at 1% *w*/*w* as the continuous phase. The beads produced were single alginate-based beads (Alg), gelling the continuous phase of the nanoemulsion, and chitosan-coated alginate beads (Alg-Ch).

The encapsulation method was based on that described by Atencio et al. [26], with some modifications used previously [8]. The nanoemulsion entered under a pressure of 400 mbar in a nozzle with a diameter of 450 μm. The sample was sprayed out using a vibration frequency of 40 Hz with an electrostatic field of 350 V to promote the separation of newly formed beads when the sample dropped into a 1.0% (*w*/*v*) CaCl_2_ solution for gelation (formation of beads), while stirred at 200 rpm. The beads were left under agitation for 10 min to allow the gelation process. They were then removed, filtered and washed with MilliQ water to obtain the Alg beads. To obtain the Alg-Ch coated beads, the Alg beads were immediately incubated using the method described by Chew et al. [45], in which they were immersed for another 10 min in a 0.1% *w*/*w* aqueous solution of Ch, and were subsequently filtered and washed.

In order to obtain Alg-Ch-TPP double gelled beads, some of the Alg-Ch beads were then immersed in a TPP solution at 5% *w*/*w*.

#### 3.7.2. Encapsulation in a Chitosan Matrix

Nanoemulsions with 30% *w*/*w* of cinnamon in the oil mixture and 2.5% *w*/*w* of oil mixture were prepared using the PIT method. The final composition of nanoemulsions was set to 2.5% *w*/*w* of oil mixture, 10% *w*/*w* of surfactant mixture and 87.5% *w*/*w* of chitosan aqueous solution at 1% *w*/*w* as the continuous phase. Due to the high viscosity of the chitosan solution, the Buchi Encapsulator could not be used. Therefore, the beads were prepared by dropping the chitosan solution into the 5% *w*/*w* TPP solution with a hand pipette. All the chitosan beads were left in the TPP solution during 10 min to assure a proper gelation. Some of the beads were reserved as single Ch beads, and the rest of the beads continued the second coating process. These beads were incubated in an alginate 1% *w*/*w* solution for 10 more minutes. Some of the resulting beads were set apart and named Ch-Alg beads, and the rest were submerged into a new 1% *w*/*v* CaCl_2_ bath for 10 min in order to gel the shell alginate coating (Ch-Alg-CaCl_2_ beads).

### 3.8. Bead Size Determination

One hundred beads produced from each type (Ch, Alg, Alg-Ch, Ch-Alg and Ch-Alg-CaCl_2_) were measured under the Optika Microscopes ST-40-2LR optical microscope (Optika, Ponteranica, Italy). The software used by all determinations was the Optika Vision Pro 3.0, Ponteranica, Italy) provided by the manufacturer.

### 3.9. Encapsulation Efficiency

To evaluate the encapsulation efficiency, all the beads were washed with the same weight of water, i.e., 20 g of beads were filtered and washed with 20 g of water. For Alg-based beads, the CaCl_2_-filtered solution and washing water were mixed. For Ch-based beads, the TPP-filtered solution and washing water were mixed. The non-encapsulated remaining cinnamon in the solution was measured using UV-VIS spectrophotometry according to the analytical techniques used by other authors [46,47,48]. The wavelength was previously studied by scanning a pattern cinnamon oil 1% *w*/*w* aqueous solution. The maximum absorbance peak was located at 279 nm.

(1) $$Encapsulation\;efficiency\left(\%\right)=\frac{Mo\;cin-M\;cw}{Mo\;cin}\times 100$$

where M_0 cin_ is the total cinnamon mass present in the nanoemulsion, and M_cw_ is the remaining mass of cinnamon in the mixture of the hardening solution and the washing water after the washing step.

The beads loading capacity was also calculated by the following equation:

(2) $$Loading\;capacity\left(\%\right)=\frac{Mo\;cin-M\;cw}{Microsphere\;mass\;sample\;(g)}\times 100$$

### 3.10. SEM Measurements

The samples were freeze dried using a Christ freeze dryer equipment model Alpha 2-4 LD Plus for 24 h and, afterwards, the surface morphology of the beads was observed using a TM 4000 Plus Hitachi SEM microscope. Characterization was performed in the Nanostructured Liquid Characterization Unit at the Institute of Advanced Chemistry of Catalonia (IQAC), which belongs to the Spanish National Research Council (CSIC) and is affiliated with the NANBIOSIS ICTS of the Biomedical Networking Center (CIBER-BBN).

### 3.11. Release Kinetics of Cinnamon Nanoemulsions Loaded in Different Beads

The release kinetics of all the produced beads was analyzed using 0.5 g of each type of bead, which was put in 200 mL of buffer solution (pH 2 or 7). The system was stabilized at 25 °C with mild magnetic stirring (50 rpm). Then, 2 mL of supernatant was extracted regularly. Subsequently, 2 mL of buffer solution with the same pH was added to the corresponding system to replace the extracted volume. The cinnamon oil was detected at 279 nm, as previously explained by UV spectrophotometry.

The release kinetics of cinnamon oil in Alg, Al-Ch, Ch, Ch-Alg and Ch-Alg-CaCl_2_ beads were analyzed using the Korsmeyer-Peppas model (Equation (3)).

(3) $$\frac{M_{t}}{M_{\infty }}={k_{p}t}^{n}$$

where k_p_ is a constant and n is the release exponent. For 0 < n < 0.45, the release regime could be described as a hindered Fickian diffusion; for n = 0.45, it could be described as Fickian diffusion; and, for 0.45 < n < 1, an anomalous transport occurred [49,50].

## 4. Conclusions

Nanoemulsions were obtained by the two low-energy emulsification methods, phase inversion temperature (PIT) and phase inversion composition (PIC), with the cinnamon oil/olive oil-F-201 surfactant–water system. The HLB temperature of the system was calculated to be 45 °C by transmittance measurements. The phase diagram was studied at the HLB and at the final temperature (25 °C). It was observed that, although there was still a small fraction of a second phase in the system at the HLB temperature and, therefore, a single phase was not obtained, the two phases present at this temperature were a bicontinuous and direct microemulsion. Therefore, at the HLB temperature, all the oil was incorporated in the structure and no free oil appeared. As a result, the formation of nanoemulsions by the PIT method was possible by a rapid change of curvature that trapped the already incorporated oil. For both methods, PIT and PIC, nanoemulsions with a very small mean diameter (4–14 nm) were obtained, but it was observed that the nanoemulsions formed by the PIT method could incorporate more % *w*/*w* of oil in their dispersed phase and, moreover, present better stability values. The nanoemulsions were gelled by adding a polysaccharide, alginate or chitosan to the continuous phase before the formation of the nanoemulsion and, once formed, dropping it into a proper gellant agent solution. These beads were covered with a layer of another polysaccharide, thus obtaining Alg-Ch beads and Ch-Alg beads, and finally an attempt was made to double gel the beads. In the case of the Alg-Ch capsules, the outer layer could not gel because the Ch-TPP bonds were very strong and dragged the Ch molecules, breaking the beads. In the case of the Ch-Alg-CaCl_2_ beads, this second layer could be properly gelled. The SEM images show how Alg beads and Ch beads have a porous structure, which causes a rapid release of the active ingredient causing a burst effect, while when adding the second layer, these pores were hidden and a slower release was obtained. In the case of the Ch-Alg-CaCl_2_ beads, it was observed how the porosity of the beads decreased noticeably and the release was more sustained, following a Fickian diffusion model with coefficients *n* of the Korsmeyer-Peppas release model of 0.40–0.45. The release profile of cinnamon oil in this case was the slowest profile; however, over time, it released almost the same amount as the other beads, indicating a more controlled and sustained release.

## Figures and Tables

**Table 1:** Kinetic parameters of cinnamon oil release from different beads at different pHs.

			Korsmeyer-Peppas Model		
Sample		M_24h_ (%)	K	n	R^2^
pH 2	Alg	77.7	0.2602	0.1664	0.9014
	Alg-Ch	72.2	0.0899	0.3075	0.9786
	Ch	78.0	0.1456	0.2462	0.9958
	Ch-Alg	59.6	0.0570	0.3311	0.9783
	Ch-Alg-CaCl_2_	55.9	0.0233	0.4485	0.9897
pH 7	Alg	78.5	0.2478	0.1870	0.8735
	Alg-Ch	71.3	0.1172	0.2670	0.9440
	Ch	76.2	0.1256	0.2554	0.9827
	Ch-Alg	65.4	0.0569	0.3580	0.9253
	Ch-Alg-CaCl_2_	55.0	0.0339	0.3937	0.9668

## Data Availability

Data are contained within the article.
